# Alteration in Activity Patterns of Cows as a Result of Pain Due to Health Conditions

**DOI:** 10.3390/ani12020176

**Published:** 2022-01-12

**Authors:** Eva Mainau, Pol Llonch, Déborah Temple, Laurent Goby, Xavier Manteca

**Affiliations:** 1 AWEC Advisors SL, Ed. Eureka, Parc de Recerca de la UAB, Bellaterra, 08193 Barcelona, Spain; 2 Department of Animal and Food Science, Autonomous University of Barcelona, Bellaterra, 08193 Barcelona, Spain; pol.llonch@uab.cat (P.L.); deborah.temple@uab.cat (D.T.); xavier.manteca@uab.cat (X.M.); 3 Boehringer Ingelheim Vetmedica GmbH, Binger Str. 173, 55216 Ingelheim am Rhein, Germany; laurent.goby@boehringer-ingelheim.com

**Keywords:** cow, pain, activity, lying time, welfare

## Abstract

**Simple Summary:**

There are several conditions and diseases considered painful to cattle. One reason for the inconsistency in pain recognition and thus pain relief in cattle is the inadequate ability to identify and assess pain. In fact, both increased and/or reduced daily lying time can be indicative of pain in cattle. This review helps to properly interpret pain in cows through behavioural activity patterns and explores whether pain relief is capable to restore their normal activity.

**Abstract:**

The main conditions and diseases considered painful in dairy cows are mastitis, lameness, calving (including dystocia and caesarean section) and metritis. The cattle literature reports that deviation from normal daily activity patterns (both increased and/or reduced daily lying time) can be indicative of painful conditions and diseases in cows. This narrative review discusses on how pain due to several health conditions in dairy cows modifies its activity pattern and explores if non-steroidal anti-inflammatory drugs (NSAIDs) are capable of restoring it. Divergent outcomes may differ depending upon the painful cause, the severity and the moment, and consequently its interpretation should be properly explained. For instance, cows with clinical mastitis reduced their time lying and increased the number of lying bouts and stepping due to pain caused by the swollen udder when cows are lying. However, lame cows show longer lying times, with a lower number of lying bouts and longer and more variable lying bouts duration, as compared to non-lame cows. When the relationship between painful disorders and daily activity patterns is studied, factors such as parity, bedding type and severity of disease are important factors to take into consideration. The potential benefits of the NSAIDs treatment in painful health disorders depend upon the type of drug administered, its dosage and administration mode, and the time of administration relative to the painful health disorder. This narrative review can be used as a tool to properly interpret and grade pain in cows through behavioural activity patterns and proposes directions for future investigations.

## 1. Introduction

Recent reviews have focused on the assessment and alleviation of pain in management procedures in cattle, such as castration and/or dehorning [1,2,3]. Other reviews focused on the assessment and management of pain associated with calving [4], lameness [5] and/or surgical pain in cattle [6]. Special emphasis has been placed on pain evaluation in dairy cattle based upon pain-specific behaviours [7] and daily behavioural patterns in sick dairy cows [8]. In addition, lying time and its association with welfare in cows has recently been reviewed [9]. Although several studies that focused on health conditions used activity patterns (especially lying time) as a pain indicator, its outcome interpretation has not been previously reviewed. This narrative review discusses how pain due to several health conditions in dairy cows modifies their activity patterns and explore whether non-steroidal anti-inflammatory drugs (NSAIDs) are capable of restoring it.

This work consists of a narrative review. All searches were performed using keyword search terms (cattle or dairy cows and pain or discomfort) in Science Direct and Scopus, including all peer-reviewed scientific articles published before May 2021. The review focuses on mastitis (or udder inflammation), lameness (or hoof trimming), calving (or parturition or dystocia) and metritis. Activity was reported as lying time, number of lying bouts, lying bout duration or number of steps. Different methodologies of activity recording were considered: direct observations, video images observations and automatic sensors of activity. NSAIDs including meloxicam, flunixin meglumine, ketoprofen, carprofen and acetylsalicylic acid were considered.

## 2. The Sensory and Emotional Experience of Pain

It is generally accepted that animal welfare comprises physical and mental health [10] and includes several aspects such as absence of thirst, hunger, discomfort, disease, pain, injuries and stress, as well as the possibility to express normal behaviours [11]. As a consequence, one of the essential components of good welfare is the recognition and control of pain.

Humans and vertebrates share similar neuroanatomical structures associated with pain perception, namely, nociceptors, nociceptive pathways and processing areas in the central nervous system (CNS) [12,13]. As a consequence, it is well established that farm animals are capable of experiencing pain.

Pain is defined by the International Association for the Study of Pain (IASP) as an unpleasant sensory and emotional experience associated with, or resembling that associated with, actual or potential tissue damage [14]. By this definition, pain is a subjective experience which requires the activity of structures in the brain to be perceived. This is in contrast to nociception, which is defined as the encoding and processing of noxious stimuli in the nervous system. Pain has a fundamentally important protective role, alerting us to threats and providing an impetus for the preservation of the integrity of the body. However, in the context of veterinary treatment of illness or injury, pain may become an unwanted consequence that is generally not useful and compromises the welfare of animals. Pain can be developed following tissue damage, inflammation and nerve injury [15]. Pain is usually transitory, lasting only until the noxious stimulus is removed or the underlying damage or pathology has healed (acute pain). However, chronic pain may persist for months and can be even more detrimental to animal welfare [16]. Chronic pain is characterized by hypersensitivity to potentially painful stimulation and is clinically manifested as spontaneous pain (pain in the absence of any stimulation), as well as hyperalgesia (an exaggerated response to a noxious stimulus) and allodynia (the presence of a pain response to a non-noxious stimulus, such as a gentle touch) [17].

Nociceptive pain may also be classified according to the site of origin (a terminology that will be used in the present review). Visceral pain results from the activation of nociceptors of the thoracic, pelvic or abdominal viscera. Visceral pain is diffuse, difficult to localize and often referred to a distant, usually superficial, structure. Superficial somatic pain is initiated by activation of nociceptors in the skin or other superficial tissue, and is sharp, well-defined and clearly located [18,19].

The pain experienced by cattle may be the result of management procedures such as castration, dehorning and tail docking, or a variety of conditions and diseases considered painful. According to different surveys of farmers and veterinarians [20,21,22,23], the main conditions and diseases considered painful in dairy cows and reviewed in the present work are mastitis, lameness, calving (including dystocia and caesarean section) and metritis. Many of these conditions occurred on the farm without the use of anaesthetics and/or analgesics for pain management [1]. Veterinarians are expected to be able to diagnose, score and treat pain in cattle. A large variability in pain scores for individual diseases and pain conditions in cows, as well as in analgesic treatment practices in cows, have been documented [21,24]. One reason for the inconsistency in pain recognition and thus pain relief in cattle is the inadequate ability to identify and assess pain [25]. In addition, economic and legislative considerations may be significant factors, too [26].

## 3. Pain Identification and Assessment in Cows

There are at least two reasons that explain why assessing pain in ruminants is particularly challenging. Firstly, as pain is a subjective personal experience, it is very difficult to objectively measure it scientifically and validate it in animals. Secondly, from an evolutionarily perspective, cattle are considered a prey species, and are inclined to avoid showing pain, even when exposed to harmful stimuli. This stoicism makes identification of pain, and therefore disease, a challenging task [27,28].

Pain indicators should be reliable (repeatable), valid and sensitive. Reliability is tested to ensure that the indicator is repeatable between (inter) and within (intra) observers and is not affected by observer bias or observer subjectivity. Validity ensures that the indicator is measuring pain and not related to any other state such as fear, anxiety or general sickness behaviour. A pain indicator should be sensitive, meaning that it allows the detection of different levels of pain and that it increases linearly with the severity of pain [29]. In addition, indicators for on-farm use should be readily applied, inexpensive, non-invasive and provide immediate (not retrospective) information [30].

Ideally, pain indicators should be validated by a “gold standard”. In humans, the self-reporting of pain has been the most commonly used method of pain assessment and validation both in clinical practice and research [31,32]. Perceived pain in animals cannot be directly measured or based upon other measures; it can only be inferred [32]. Since validation is based upon the measurement of an indicator against a “gold standard”, pain indicators must be thoroughly evaluated using experimental studies. The majority of studies used several measures simultaneously, with different experimental approaches, to evaluate the potential value of these measures as indicators of pain [32]. Pain assessment in animals has tended to use four approaches: measures of general indices, physiological indicators, behavioural indicators [28] and, recently, facial expressions [33]. Some studies integrate those measures into a graded scale in order to fully assess the impact of a painful condition or event on the individual (e.g., [7]). Pain assessment based on behaviour has received increasing attention and is the most commonly used parameter to assess pain in farm animals [34]. Three types of behaviours, useful for pain evaluation in farm animals, have been proposed [7,28]: (1) pain-specific behaviours; (2) avoidance-of-pain behaviours; and (3) a change in certain behaviours that the animals are very motivated to perform.

Pain-specific behaviours encompass a promising method of assessing pain in farm animals (e.g., [30]). This approach has been used extensively in rodents to assess pain in order to provide the best possible treatment and refine painful techniques [35]. For instance, decreased burrowing behaviour and voluntary wheel running, weight bearing/gait, abnormal postures, paw flinches and ultrasonic vocalizations have been systematically observed in multiple rat models of chronic pain [36]. However, pain-specific behaviours are usually obtained by direct and continuous observations by trained observers (e.g., [7,29]), thus making it time-consuming.

Avoidance-of-pain behaviours assess the reaction of animals upon being manipulated and is a commonly used method to assess pain. These indicators are considered valid and reliable as long as the reaction is scored in a standardized way [37]. Pain sensitivity has been quantified using mechanical (e.g., [38]) or thermal stimulation of a hind leg or the udder (e.g., [39]), mainly to assess pain in cows suffering from mastitis. These methods measure the nociceptive threshold, defined as the minimum stimulus necessary to elicit a pain response. When a stimulus is applied to a painful site, a cow responds with avoidance behaviour such as kicking, leg lifting or tail flicking [40]. Lower nociceptive threshold values indicate that there is increased pain.

A change in certain normal behaviours, such as daily activity patterns, including lying behaviour, has been used extensively in the cattle literature to assess pain caused by several conditions. However, up to now, behavioural activity patterns have not been properly interpreted as a pain indicator in cows. Several electronic data loggers are widely available and can be used to measure lying behaviour accurately, including the total time spent lying down, the number of lying bouts and the duration of each bout for individual cows [41]. The automatic recording of behavioural activity (lying, standing and walking) can be achieved using a variety of sensor systems; for example, mercury tilt switches, three-dimensional accelerometers embedded sensor technology and automatic local position systems [42,43].

Daily activity patterns indicative of pain in cows have been investigated in experimental studies by (a) animals as their own control: observing animals before, during and after a painful process or procedure; (b) comparing animals thought to be in pain to the controls (pain-free animals); (c) observing animals thought to be in pain and receiving effective analgesia, as compared to a placebo; and (d) observing whether indicators of pain increase with the severity of pain. Ideally, a combination of these methods would be used to test the validity of behavioural pain indicators [30].

The main behavioural indicator of activity patterns used in cows is the time spent lying down. Other related behavioural indicators of activity patterns used are the frequency of lying bouts (i.e., a transition from standing to lying), the duration of individual bouts and activity or number of steps. Healthy lactating cows lay down 11.0 ± 2.1 h/d in 9 ± 3 bouts/day, with a bout duration of 88 ± 30 min/bout. These values ranged from 9.5 to 12.9 h/d, 7 to 10 bouts/d and 65 to 112 min/bout across farm means [44]. However, lying behaviour varies considerably among dairy systems, with the shortest duration often in pasture systems and the longest usually in tie-stalls (see [9] for further revision).

Lying is a behavioural need for dairy cattle. Lying is considered to be a higher priority than eating and social contact when opportunities to perform these behaviours are restricted [45]. As a consequence, lying behaviour has been identified as an element that can be used to measure a cow’s welfare status [45,46,47]. Lying behaviour has been identified as a sensitive measure of animal comfort (e.g., [48]) and to assess pain caused by several conditions in cows (e.g., [49]). Deviation from normal lying behaviour (both increase or reduction) can be indicative of pain in cows. Longer lying times (within a normal range) are generally indicative of increased welfare [9]. However, resting for a long time with few lying bouts can also indicate illness. Sickness behaviour is a well-organized adaptive response to enhance disease resistance and facilitate recovery from disease [50]. Sickness behaviour includes changes in behaviour and physiology. In fact, tissue injury and infection unleash the signals necessary for immune activation. Inflammatory mediators released during immune activation are initiated by a group of chemical mediators known as cytokines [51]. Interleukins are a particularly important group of compounds within cytokines and they mediate the acute-phase response by initiating fever, synthesis of acute-phase proteins and the immune response originating in lymphoid tissue, among others [51]. Beside this, they act on the brain (hypothalamus), which initiates anti-inflammatory mechanisms and some of the main behavioural effects involved in sickness behaviour. Sickness behaviour can include lethargy, apathy, somnolence, fever, loss of appetite and thirst, reduced social interaction and a decrease in general physical activity, among other symptoms. Lying is often increased in ill animals [50,52], where reduced activity is considered beneficial for energy conservation and maintenance of the febrile response [53].

Overall, in order to properly understand how a painful condition can affect lying behaviour in cows, different aspects have to be considered. Firstly, the origin, location, intensity and source of pain have to be identified. Secondly, irrespective of pain, it is necessary to identify if the cow shows some or several symptoms associated with sickness behaviour.

Daily activity patterns are affected by different factors others than pain. Studies assessing pain in cows should standardize other factors affecting daily activity patterns or at least consider them as influencing factors. Daily activity patterns can be influenced by individual, environmental, housing system and management factors. The main individual factors described are parity [54], social ranking [55], days in milk (DIM) [56] and milk yield [48]. The main environmental factors described are seasonal changes [57,58], including heat stress [59]. The main housing-system factors described are dairy systems [60,61,62,63], stall dimensions [64], stall features [65,66], stall surface [67] and bedding material [68]. The main management factors described are stocking density [69,70,71], grouping strategies [72], feeding management [73] and time spent away from the pen for milking [65]. In summary, other factors are known to have an important effect on daily activity patterns (especially lying behaviour). Clearly, these factors have to be taken into consideration when an experimental study assessing pain through daily activity patterns is done and caution is required in extrapolating findings to other conditions.

## 4. Pain Management in Dairy Cows

Pain relief is based on treating inflammation and other systemic effects that commonly accompany inflammation and slow any further tissue damage. The use of corticosteroids may not be appropriate because of their immunosuppressive and metabolic side effects [74,75]. Numerous NSAIDs are available and licensed for use in farm animals, demonstrating their safety and efficacy. Over the last decade, there has been an increase in their use, probably because there is increasing evidence to support the benefits in painful diseases such as lameness, mastitis and metritis [26]. NSAIDs are commonly used in animals to reduce inflammation (anti-inflammatory), reduce pain (analgesic) and decrease overall body temperature (antipyretic). Incorporating NSAID therapy into treatment protocols for a variety of painful conditions should improve the welfare of animals and, consequently, decrease the related economic losses [40,76]. When a NSAID is administered prior to a specific painful condition or disease (instead of administering NSAIDs during or after the painful moment), animals returned to a normal physiologic state more quickly [77]. NSAIDs act by inhibiting the cyclooxygenase enzyme (COX), which in turn prevents prostaglandin synthesis [40]. It is known that while COX-1 acts mainly on housekeeping physiological functions, COX-2 is normally activated in specific conditions such as inflammation [78]. Therefore, the therapeutic effect of NSAID drugs is mostly derived from their ability to inhibit COX-2, while most of their side-effects (gastrointestinal irritation, renal toxicity and inhibition of blood clotting) are caused by the inhibition of COX-1 [79]. The most frequently cited NSAIDs used for painful conditions in cattle are flunixin meglumine, meloxicam and ketoprofen [1,21,80]. Meloxicam is known to be a preferential COX-2 inhibitor [81], having a targeted action against inflammatory processes and optimal safety profile in cattle. Both ketoprofen and flunixin meglumine have a prevalent activity on COX-1. The half-life elimination of flunixin meglumine is 3 to 8 h, and ketoprofen has a short half-life of 0.42 h, and therefore requires extra dosing. In turn, the elimination half-life of meloxicam is found to be 24–26 h [82]. Other NSAIDs used in cattle are salicylate (COX-1 and -2) and carprofen (COX-2 selective and well-tolerated in cattle) [83]. Furthermore, it is important to note that with most NSAID treatments, milk discarding is mandatory for at least 24 h to prevent contamination with drug residues (see [83] for further review).

## 5. Pain Associated with Mastitis

Mastitis is an inflammation of the mammary gland that may be caused by a number of different bacteria. Mastitis is a multifactorial disease in which the environment, the pathogens and the host (cow) interact with each other. Despite being a major animal welfare and economic problem in dairy cattle production [84], mastitis is usually underestimated by farmers [85]. The incidence rate of clinical mastitis ranges from 13 to 40 cases/100 cows per year in different countries and housing types [84].

From several surveys performed using farmers and veterinarians, mastitis due to *Escherichia coli* (*E. coli*) in cows was given a high score for pain (from 7 to 9 out of 10). However, mild mastitis (milk clots only) received lower painful scores (from 1 to 3 out of 10) [21,24,86]. During clinical mastitis, the concomitant inflammation of the udder, increased intramammary pressure and increased external pressure (e.g., of the adjacent limb on a swollen udder) induce pain [40]. It is accepted that cows can also experience pain in mild or moderate cases of clinical mastitis. Even subclinical mastitis is accompanied by increased levels of bradykinin, a peptide that mediates the inflammation related to mastitis [87]. Additional knowledge on the levels of pain experienced by cows, depending on the phase of the disease (especially during the remission phase), is needed. In dairy cows, after udder inoculation with *E. coli*, de Boyers des Roches et al. [88] suggested that cows may have experienced discomfort in the pre-clinical phase (0–8 h post-inoculation) and pain in the acute phase (12–24 h post-inoculation), but neither discomfort nor pain in the remission phase (32–80 h post-inoculation). Nevertheless, Fogsgaard et al. [89] showed in dairy cows with naturally occurring mastitis, that local clinical udder signs as well as behavioural changes persisted throughout one week after antibiotic treatment. Severe cases of mastitis lead to hyperalgesia and allodynia [90]. A hyperalgesic state lasting at least four days has also been described in cows with mild or moderate mastitis [91].

### 5.1. Alteration in Activity Patterns as a Result of Pain Due to Mastitis

Although lying time usually increases during illness and it is thought to help the animal to save energy, most studies observed that cows with clinical mastitis (experimentally induced with endotoxin or *E. coli* or naturally occurring) reduced their time lying and increased stepping (Table 1). The most likely explanation for the reduced lying time is pain caused by the swollen udder when cows with mastitis are lying [92,93]. It may be possible that lying through direct contact with a hard surface causes more pain to the udder than having the udder hanging freely while standing [94]. In fact, the cows increased their standing time simultaneously with the local swelling of the udder quarter [92], and time spent lying was negatively correlated with rectal temperature and blood cortisol during the first 12 h after intramammary infusion [95]. Moreover, the distance between the hocks when the cow is standing is wider in cows with mild and moderate mastitis, when compared to healthy cows, suggesting that there has been a change in the hindleg stance of the cows as a result of the inflamed udder [96]. If lying is painful for the cow, and thus reduced, the cow might become increasingly frustrated and restless over time. The increased stepping and increased frequency of lying bouts [89] may be indicative of such motivational conflict and, hence, of coping difficulties in response to the disease. The increased stepping may be also a pain-avoidance behaviour [97], as an altered stance has earlier been shown in cows suffering from mastitis [96].

Recent descriptions of altered behaviour during experimentally induced clinical mastitis in cows have shown normalization within a few days after diagnosis [92,93]. However, Fogsgaard et al. [89] reported that clinical mastitis occurring naturally might last at least 10 days (one week after antibiotic administration).

Nonetheless, the differences among activity measurements observed after a *Streptococus uberis (S. uberis)* challenge in cows (increased lying time and reduced stepping) reflect a difference in host mammary responses to different pathological agents [98]. Similarly, other studies did not show differences in the activity or resting behaviours, presumably because other factors such as stage of lactation or housing conditions (free-stalls vs. tie-stalls) are not completely comparable.

In addition, some authors observed that cows with mastitis spent [93] or tended to spend [92] less time lying on the side of the affected udder quarter. Pain or discomfort when lying on the affected udder quarter would cause the cow to spend more time lying on the other udder side, as an avoidance behaviour. Other studies did not report any difference in lying time depending on the affected udder side [94,99,100,101], suggesting that cows seem to have a preference as to which side they lie upon, regardless of the infection status of their mammary gland [100].

### 5.2. The Effect of NSAIDs on Activity Patterns in Cows with Mastitis

In general, the use of NSAIDs has been shown to decrease rectal temperatures, decrease signs of inflammation, restore rumen motility, increase the eating time and dry-matter intake and reduce heart rates in experimentally induced mastitis cases, as compared with their untreated counterparts [27]. However, the amount of time cows spent lying was not affected by flunixin meglumine following experimentally induced mastitis [95,100]. It can be expected that NSAIDs would reduce udder inflammation and increase the lying time. The reason why flunixin meglumine did not show these expected effects remains unclear. Several factors, such as the short half-life elimination of flunixin meglumine [83] or delayed administration after challenge (from 4 h to 24 h) [95,100], could have influenced the result. Additional research including the effect of other NSAIDs on experimentally induced mastitis on lying time should include a closer administration before or after challenge and/or a repeated dose administration. To our knowledge, no study reports the effect of NSAIDs on lying time in naturally occurring mastitis in dairy cows.

**Table 1:** Summary of changes in activity patterns in cows (lying time, lying time on quarter affected, number of lying bouts, lying bout duration and activity) caused by mastitis. Studies are summarized according to how they have been assessed in relation to pain using the following methods: cows studied as own control (before, during and after mastitis) and comparing cows with mastitis to the control. Moment (in days, d, or hours, h, related to challenge or naturally occurring mastitis; D0 = day of challenge or diagnosed natural mastitis) and type of change (↑ increase; ↓ decrease; NS = non-significant; NE = non-evaluated) are given comparing the painful moment or cows in pain due to mastitis versus the control moment or control cows.

Studies Classification	Reference	Origin of Pain Due to Mastitis	Control Moment or Control Cows	Lying Time (h/d)	Lying Time on Quarter Affected (h/d)	No. Lying Bouts (No./d)	Lying Bout Duration (min/d)	Activity (No. Steps/Day)
Cows as own control (before, during and after mastitis)	[94]	Challenge (D0) with *E. coli*	Prechallenge (D-1, D-2)	↓ (from +4 h to +7 h)	NS	NS	NS	NE
			Postchallenge (D+1, D+2)	NS	NS	NS	NS	NE
	[92]	Challenge (D0) with *E. coli*	Prechallenge (D-1)	↓ until +20 h	↓ (t)	NS	NS	↑
	[93]	Challenge (D0) with *E. coli*	Prechallenge (D-1, D-2)	↓ (D+2)	↓ (D+2)	NE	NE	NE
			Postchallenge (D+1, D+2)	↓ (D+2)	↓ (D+2)	NE	NE	NE
Mastitis vs. control	[95]	Challenge with *E. coli* + PBS 4 h post-challenge	Intrammamary infusion with PBS + PBS 4 h post-challenge	NS (from 0 h to +24 h)	NE	NE	NE	NE
	[100]	Challenge with *E. coli* + PBS	Non-challenged: Infusion with PBS	↓ (from +3 h to +6 h)	NS	NS	NS	NS
	[98]	Challenge with *S. uberis +* PBS	Uninfected	↑ (D+4)	NE	NS	NE	↓ (from D+1 to D+3)
	[89]	Cows with naturally occurring clinical mastitis	Clinically healthy control cows	↓ (from D0 to D+2)	NE	↑ (from D+3 to D+10)	NE	↑ (from D+6 to D+10)

Significant differences are established at *p* ≤ 0.05, and tendency (t) at *p* ≤ 0.1. PBS = phosphate-buffered saline (water-based salt sterile solution that helps to maintain a constant pH).

## 6. Pain Associated with Lameness

Lameness is characterized by an abnormal gait caused mainly by an injury, a disease, or a dysfunction of one or more feet and/or limbs. Lameness is a multifactorial disease associated with management, equipment features, nutrition and genetic selection hazards [102]. Lameness compromises the welfare of affected animals and results in economic losses due to reduced milk yield and fertility and increased treatment cost [103]; however, lameness is usually underestimated by farmers [104]. Under-recognition of lameness occurs in all cases, slight lameness being the most affected. The major effect of this under-recognition of lameness is not that lame cows are not treated but that treatment is delayed more than 3 weeks, especially of less severely lame cows [105]. Lameness prevalence ranges from 5.1% to 54.8% [106], with the highest rates observed in herds housed in tie-stalls [107] and free-stalls [108,109,110]. Although the effect between free-stall and tie-stall housing on lameness incidences is not clear [107], several studies conclude that lameness in tie-stalls system are underestimated due to the difficulty of assessing it [111,112]. The use of a Lameness Scoring System (LCS) on a regular basis is the most effective means of identifying lameness in cows. A LCS describes the gait properties to classify the severity of lameness on a numerical scale [104]. The five-point LCS is one of the most frequently employed methods in lameness detection (score 1 = normal; score 2 = mildly lame; score 3 = moderately lame; score 4 = lame; and score 5 = severely lame) [113].

Several surveys performed using farmers and veterinarians concluded that different diseases in cows that can lead to lameness are scored as a painful condition. For instance, interdigital necrobacillosis, digital dermatitis and swollen hock received the following painful scores on a scale of 10: 8.2, 7.0 and 5.3, respectively, by farmers, and 7.5, 6.3 and 5.4, respectively, by veterinarians [23]. In another survey, Canadian veterinarians scored acute lameness as 6.4 and chronic lameness as 5.2 on a scale of 10 [20]. Claw lesions remain among the major causes of lameness in dairy cows. Injuries of the claws or the limbs cause painful bruises, which lead the cow to change its locomotion due to pain avoidance. Tissue damage or infections of the claw are enough to induce sickness behaviour (e.g., reduced feed intake and lethargy), as inflammatory cytokines are released. Furthermore, non-inflammatory reasons for lameness lead the cows to change their behaviour independently of inflammatory cytokines [8,114]. Hyperalgesia has been observed at the time of lameness detection and one month later, suggesting that clinically lame cows suffer from chronic pain [5]. Furthermore, cows with moderate lameness can also experience pain and pain can appear before the cow becomes clinically lame [115].

### 6.1. Alteration in Activity Patterns as a Result of Pain Due to Lameness

Most of the studies report that lame cows have longer lying times, with a lesser number of lying bouts and longer and more variable lying bouts duration, as compared with non-lame cows (Table 2). For example, Solano et al. [116] showed that cows with a lying time ≥ 14 h per day, ≤5 lying bouts per day, bout duration ≥ 110 min/bout or standard deviations of bout duration over 4 d ≥ 70 min had 3.7, 1.7, 2.5 and 3.0 odds of being lame, respectively. Lame cows are less active (in terms of number of steps per hour) than non-lame cows [117], whereas Chapinal et al. [118] did not find this difference. Similarly, hoof trimming (a preventive procedure that reduces the incidence of lameness) also provokes increased time spent lying on the day of hoof trimming, and for up to 4–5 weeks afterwards, and a reduced activity on the day of hoof trimming in both lame and non-lame dairy cows [119,120,121,122].

The severity of foot lesions (mild, moderate or severe) had a more pronounced effect on reducing the daily activity levels of cows than did the type of lesion present (digital dermatitis, sole ulcer or white-line disease). In fact, increased time spent lying down is associated with increased locomotion scores [123], and increased locomotion scores are associated with increased weight-shifting behaviour, a marker of pain in lame cows [118]. Severely lame cows present a longer daily lying time and lying bouts duration than do moderately lame cows [23,114,123]. Lesions that cause the greatest increase in lying behaviour are digital dermatitis and sole ulcers, which have longer lying bouts duration and, consequently, spend more time lying down per day [23,124]. For instance, cows with sole ulcers spent > 1 h/d more lying down, and their mean lying bout duration is 20 min longer, when compared to cows without hoof lesions [119].

These findings suggest that the pain associated with ulcers may reduce the willingness of a cow to stand up once she is lying down [124]. Tissue injury and infection, which are common elements of lameness in dairy cows, lead to immune system activation and the behavioural modifications intrinsic to sickness response. In fact, in addition to changes in locomotion and activity, lame cows display decreased food intake and altered social behaviour, such as increased self-grooming behaviour [125]. Nevertheless, lying time per se does not seem to be a potentially useful indicator for detecting moderate lameness in dairy herds [126].

Although most studies report an increase in lying time in lame cows, Cook et al. [67] report a decrease in lying time in lame cows allocated in free stalls with sawdust. Other studies report no differences or a tendency of an increase in lying time for lame cows [123,126]. A potential reason for the lack of association between lying time and lameness could be that severely lame cows are not included in some studies (e.g., [126]). In addition, in lame cows, lying behaviour is specially affected by bedding type. For instance, lame cows spent more time standing in stalls than did non-lame cows, but this difference was greater with mattress stalls, as compared with sand stalls [67,127]. Severely lame cows in deep-bedded stalls lie down longer than do non-lame cows, whereas in mattress herds, no difference in lying time was observed [123]. Similarly, moderately lame cows had a reduced lying time, when compared with non-lame cows in herds with a mattress, an effect not observed in herds with sand [68]. In summary, increased lying time due to lameness may be more difficult to identify in mattress stalls, whereas lame cows in bedded stalls are more prone to maintain their normal resting behaviour. Clearly, when the relationship between lameness and lying behaviour is studied, bedding material and the inclusion of severely lame cows are important factors to take into consideration, and special caution is required in extrapolating findings to other studies [118]. In addition, high variability between lying time in cows [44] could hinder finding differences between lame and non-lame cows using activity measures alone. Lame cows show contradictory extreme lying times, suggesting that the effect of lameness on lying behaviour must consider the pain suffered in both rising and lying movements. The interaction with the surface during both rising and lying movements may appear as difficult (and painful) in lame cows. Some lame cows may find it difficult (and painful) to stand, and thus lie down for longer (in total and per each lying bout), whereas others may find it difficult (and painful) to lie down, and thus show a lower number of lying bouts and stand in the stall for longer [68].

**Table 2:** Summary of changes in activity patterns in cows (lying time, number of lying bouts, lying bout duration, standard deviation of bout duration and activity) caused by lameness. Studies are summarised by comparing cows with lameness to the control (including degree of severity). Lameness assessment score (from 1 = non-lame to 3 or 5 = severely lame) and housing system and bedding type are documented. Type of change (↑ increase; ↓ decrease; NS = non-significant; NR = non-reported; NE = non-evaluated) is given comparing cows in pain due to lameness versus the control cows.

Reference	LSC	Criteria of Painful Moment or Cows in Pain Due to Lameness	Criteria of Control Moment or Control Cows	Housing System and Bedding Type	Lying Time (h/d)	No. Lying Bouts (No./d)	Lying-Bout Duration (min/d)	SD of Bout Duration (min)	Activity (No. Steps/h)
[116]	5-point scale	Score ≥ 3	Score 1 and 2	Free-stalls (different types of bedding)	↑	↓	↑	↑	NE
[68]	4-point scale	Score 2	Score 1	Free-stalls (mattress + sand)	NS	↓	NS	NE	NE
		Score 3	Score 1		↑ (mattress) NS (sand)			NE	NE
[126]	5-point scale	Score 3 or 4	Score 1	Free-stalls (straw)	NS	NS	↑	NE	NE
[67]	3-point scale	Score 2 or 3	Score 1	Free-stalls (mattress + sawdust)	↓	NE	NR	NE	NE
		Score 2 or 3	Score 1	Free-stalls (mattress + sand)	NS	NE	NR	NE	NE
[127]	4-point scale	Score 3	Score 1 and 2	Free-stalls (mattress + sawdust; and sand)	↓	NE	NE	NE	NE
[124]	5-point scale	D: score = 3.0 H: score = 2.7 U: score = 3.3	NL: score = 2.9	Free-stalls (sand)	D: NS H: NS U: ↑	D: NS H: NS U: ↓	D: NS H: NS U: ↑	NE	NE
[118]	5-point scale	Score > 3	Score ≤ 3	Free-stalls (sand)	↑(t)	NS	↑	NE	NS
[128]	4-point scale	Score 2 or 3 Trimming	Score 0 or 1	NR	NS	NS	NS	NE	NE
		Score 2 or 3 Trimming + foot block	Score 0 or 1	NR	↑	NS	NS	NE	NE
[123]	5-point scale	L: score ≥ 3 SL: score = 4	NL: score ≤ 2	Free-stalls (deep-bedded)	L: ↑ (t) SL: ↑	L: NS SL: NS	L: NS SL: ↑	L: NS SL: ↑	NE
				Free-stalls (mattress)	L: ↑ (t) SL: NS	L: NS SL: NS	L: NS SL: NS	L: NS SL: ↑(t)	NE
[117]	5-point scale	Score ≥ 3	Score ≤ 2	Free-stalls (bedding not described)	NE	NE	NE	NE	↓

Significant differences are established at *p* ≤ 0.05, and tendency (t) at *p* ≤ 0.1. SD = standard deviation; LSC = Lameness Scoring System; D = dermatitis; H = haemorrhages; U = ulcers; NL = non-lame; L = lame; SL = severely lame.

### 6.2. The Effect of NSAIDs on Activity Patterns in Cows with Lameness

The major part of studies assessing the effect of NSAIDs on lameness and lying behaviour are field trials performed around the moment of hoof trimming (Table 3). Flunixin meglumine has shown substantial acute analgesia in the induced lameness model, as shown through improvement of the gait score and increased pressure on mat systems on the affected foot and claw [129,130]. However, in field trials, NSAIDs have shown variable results, with a mild improvement in the locomotion score and nociceptive thresholds [5].

To our knowledge, only one study assessed the effect of NSAIDs on induced lameness in cattle (by an intra-articular injection of amphotericin B) and recorded lying behaviour as an indicator of pain relief [129]. Schulz et al. [129] reported that flunixin-treated steers spent less time lying than did their control counterparts during the early post-induction lameness. Regarding field trials, Offinger et al. [131], studying cows severely lame from septic arthritis, showed that meloxicam treatment improved gait scores after surgery. In addition, cows treated with meloxicam showed a reduced daily lying time and a higher number of steps per hour, as compared with control cows. On the contrary, other field trials showed no significant differences [118,128], only a tendency [119] or results with little clinical relevance [121] when evaluating the effect of NSAIDs on lameness and lying behaviour and/or activity. These inconsistent outcomes between induced lameness and field trials may be a result of clinical heterogeneity present in different cases of clinical lameness in field trials and/or a low sensitivity of indicators to detect analgesia effects on moderate lameness [5]. Additional studies are needed to further investigate the potential analgesia effect of NSAIDs in mitigating the pain associated with lameness, both in induced lameness models and field studies. These studies should accurately assess the degree of severity of lameness and, if possible, to identify the most plausible cause of lameness. Lying behaviour can be included as one of the measures to assess pain in lame cows, but other indicators, such as pressure mats and a locomotion score, should be included.

## 7. Pain Associated with Calving

Calving itself (and especially dystocia) is a cause of pain [4]. Dystocia can be defined as a difficult parturition resulting from prolonged spontaneous parturition or prolonged or severe assisted extraction [132]. Calving normally occurs between 30 min and 4 h from the appearance of the amnion to the birth of the calf [133]. The two main causes that can produce calving longer than normal are foeto-pelvic disproportion and foetal malpresentation. From several surveys, dystocia was ranked by veterinarians and farmers as one of the most painful conditions of cattle, obtaining a score from 7 to 8 out of 10 [21,22,86]. Most dairy cows experience a physiological subacute inflammation during the first days after calving [134]. If the degree of inflammation exceeds a certain level, it becomes harmful, impairing normal lactation [83]. In fact, around the time of birth, the levels of acute-phase proteins (such as haptoglobin and serum amyloid protein) increase considerably in response to inflammation, tissue damage and, thus, pain [135]. Injury, trauma and inflammation associated with calving (particularly during dystocia calving) can have important negative effects on health, welfare and productivity [4].

Caesarean sections in dairy cattle are carried out in approximately 1–2% of calvings [136,137]. Following a caesarean section, cows may experience pain of somatic and visceral origin, arising from the surgery itself and failed attempts at vaginal delivery [4,137,138,139]. Caesarean sections have been rated at 9 on a 10-point pain severity scale by UK veterinary surgeons [21], suggesting that pain associated with a caesarean section is a significant welfare concern. However, very little is known about the pain experienced by cattle following a caesarean section.

### 7.1. Alteration in Activity Patterns as a Result of Pain Due to Calving

The activity of cows increases quite dramatically prior to calving. On the day of calving, cows spent approximately 2 h less time lying down and spent an average of 31.4 min less per lying bout, as compared with the days before calving (e.g., [140]). The number of lying bouts were more than doubled from Day-2 to Day-1 prepartum [141]. Activity such as number of steps per day on the day of calving ranges from 2847 to 5424 steps/day [135,140] and represents an increase from 57% [135] to 62% [140], when compared to baseline days (7 days before or 7 days after calving, respectively). Although this behavioural pattern might be a part of the normal calving situation, it has been suggested that this increased restlessness may be due to pain caused by uterine pressure and cervical dilatation [4,142]. Alternatively, and in a complementary way, increased activity may also be explained by their natural instinct to distance themselves from the herd to find a secluded place to give birth [143,144].

Deviation from normal activity behaviour around calving (either increased or reduced activity) are indicative of additional pain during the calving process. Cows that delivered stillborn calves have greater prepartum activity than do those that delivered live calves, probably indicating additional pain before calving [145]. However, cows that experienced dystocia spent more time lying, had a fewer number of steps, had more lying bouts and had shorter lying bouts duration than did eutocic cows from calving to 7 DIM [146]. Similarly, cows who delivered twins have reduced activity during prepartum, as compared with cows delivering singletons [145]. In those two latter cases, the reduced prepartum activity observed in dystocic cows or cows delivering twins suggest that dystocic cows are subjected to additional pain before calving (Table 4). Primiparous cows show a higher number of steps [135,140] and reduced total time lying [49], when compared to multiparous cows around calving. Apart from the lack of experience of the dams at first parturition, the majority of studies confirm that primiparous females have longer durations of parturitions and the degree of effort associated with it is usually greater than in multiparous females [133]. In order to better correlate pain due to calving with lying behaviour, the degree of difficulty at calving should be considered. Currently, pain scales used to grade dystocia are very subjective and complicate attempts to compare or merge findings between farms and countries [147]. Future studies should incorporate pain scales more objectively at calving, for instance, including physiological indicators of inflammation to properly grade the degree of pain at calving.

Activity patterns around calving can be easily affected by other factors than pain. Such confounding factors (e.g., metabolic strain of lactation, management, adaptation to new calving environment, early calf-mother separation, etc.) can all influence the activity-patterns expression of post-partum pain [137].

### 7.2. The Effect of NSAIDs on Activity Patterns in Cows with Calving

The potential benefits of the treatment of periparturient cows with NSAIDs depend upon the type of drug employed, its dosage and administration mode, and the time of administration relative to calving. Overall, milk yield and composition are not affected by NSAIDs administration after calving. Nonetheless, the impact of NSAIDS on milk yield and composition may vary depending upon cow inflammation levels. Indeed, the administration of salicylate increases milk production of cows with high haptoglobin levels [148,149]. Positive effects of NSAIDs that preferentially inhibit COX-2 activity, such as meloxicam, on milk production seem to be plausible in a large-scale study [150]. It has been reported that NSAIDs that preferentially inhibit COX-1 activity, such as flunixin meglumine, can trigger side effects such as an increased risk of retained placenta, culling or metritis [151], reduced feed intake and increased rectal temperature after calving [152]. On the contrary, COX-2-preferential NSAIDs displayed possible benefits to health and performances such as a lower culling rate, a lower risk of mortality on the farm, a reduced incidence of mastitis and an increased pregnancy rate at first insemination [148,150,153].

According to studies carried out to date, lying time, number of lying bouts and lying bouts duration were not significantly affected by NSAIDs treatment around calving (Table 5). However, the activity (in terms of number of steps per day) was altered by NSAIDs treatment around calving, although it shows divergent results among studies. Eutocic cows treated with acetylsalicylic acid [146] and primiparous eutocic cows treated with meloxicam [135] after calving coincided in that NSAID treatment increases activity during the first 2 DIM, when compared to the controls. NSAIDs could lead to a reduction in the mediators of inflammation (cytokines and prostaglandins) responsible for the expression of sickness behaviour, such as lethargy and depression, observed in inflammatory states [154,155]. In a similar way, Newby et al. [156] reported that meloxicam administered after calving in cows increased the number of feeding visits and total time spent feeding, suggesting some pain alleviation after calving. On the contrary, Ref. [157] reported that meloxicam administered either before or after calving decreased activity post-calving in the Jersey breed (but not in Holstein) and in cows with dystocia (defined as cows that calved in >70 min). The authors speculate that administration of meloxicam to dystocic animals reduced activity by alleviating inflammation, allowing the cow to rest more easily. Similarly, Barrier et al. [137] observed that beef cows treated with meloxicam prior to a caesarean section spent more time lying and had more lying bouts following surgery, as compared to cows receiving a placebo. This last result concurs with previous research examining behaviour following abdominal surgery [137,158,159].

**Table 4:** Summary of changes in activity patterns in cows (lying time, number of lying bouts, lying bout duration and activity) caused by calving. Studies are summarized according to how they have been assessed in relation to pain using the following methods: cows studied as their own control (before, during and after calving) and comparing cows with dystocia to eutocic calving. Moment (in days, D; D0 = day of calving) and type of change (↑ increase; ↓ decrease; NS = non-significant; NE = non-evaluated) are given comparing the painful moment or dystocic cows versus the control moment or eutocic cows.

Studies Classification	Reference	Painful Moment or Cows in Pain Due to Calving	Control Moment or Control Cows	Lying Time (h/d)	No. Lying Bouts (No./d)	Lying Bout Duration (min/d)	Activity (No. Steps/d)
Cows as own control (before, during and after calving)	[135]	Around calving (from Day-1 to +2)	Post-calving (from D+3 to D+7)	NE	NE	NE	↑
	[140]	Calving day (24 h before calving)	Pre-calving (from -24 h to D-4 to D-11)	↓	↑	↓	↑
	[58]	Post-calving (D+4, D+5)	Post-calving (from D+5 to D+28)	↓	NE	NE	NE
Dystocia vs. eutocia	[146]	Calving ease score of 3 (assistance from 2 or more people) or score of 4 (mechanical traction assistance)	Calving ease score of 1 (unassisted) or 2 (assistance from 1 person)	↑	↑	↓	↓
	[135]	Easy manual pull	Unassisted	NE	NE	NE	NS
	[157]	Cows that calved in >70 min	Cows that calved in ≤70 min	NS	↑ (D0) ↓ (D+2)	↑ (D+1, D+2, D+4, D+5)	↓ (Holstein D+1, D+4, D+5 and Jersey D0, D+2)

Significant differences are established at *p* ≤ 0.05.

## 8. Pain Associated with Metritis

Clinical metritis is an inflammatory process that affects all layers of the uterus during the early postpartum period in lactating dairy cows [161,162]. This condition is characterized by the presence of an abnormally enlarged uterus with a fetid red-brownish uterine discharge, with or without systemic signs of illness such as depression, anorexia, decreased milk yield and pyrexia within 21 d after parturition [49,162,163]. The incidence of clinical metritis ranges from 15% to 20%, although it could be higher, affecting up to 40% of cows, depending on the herd and lactation stage [164,165,166,167]. Metritis has been associated with reduced milk production, impaired reproductive performance and increased risk of culling during lactation [168,169,170,171,172,173]. Besides the negative economic implications, clinical metritis is a welfare concern. From several surveys performed using farmers and veterinarians, acute metritis in cows was given from 4 to 6 out of a 10-point score for pain [21,22,24,86]. In fact, it is known that cows with clinical metritis have shown physical signs of pain such as back arching at rectal examination, as compared with cows without clinical metritis, suggesting that clinical metritis may be associated with visceral pain [174].

### 8.1. Alteration in Activity Patterns as a Result of Pain Due to Metritis

Alteration in activity patterns due to metritis appears to be more pronounced in primiparous cows than in multiparous ones. Primiparous cows diagnosed with clinical metritis spent more time lying (10.1 h/day vs. 9.2 h/day, respectively) during one week after metritis diagnosis, when compared to primiparous cows without metritis [49]. In addition, reduced activity has been reported in cows diagnosed with metritis (as measured with neck-mounted accelerometers [145,175]). In grazing dairy cows, Sepúlveda-Varas et al. [176] also found that primiparous cows diagnosed with more than one health disorder (retained placenta, mastitis or metritis) spend more time lying and tended to have longer lying bouts in the days following calving, as compared with healthy cows. When studying multiparous cows, no differences were reported in terms of daily activity patterns between healthy cows and those with metritis [49,177]. During the early postpartum period, primiparous cows show an exacerbated inflammatory response (increased haptoglobin and serum amyloid A) when compared to multiparous cows [135,178]. This suggests that the immune system of primiparous cows may be more reactive to inflammatory stimuli and may lead to an increase in circulating cytokines. As cytokines play a key role in the sickness behaviour, the increased time lying associated with clinical metritis should be more evident in primiparous cows than in multiparous ones [49].

Metritis seems to affect a cow’s activity pattern before metritis gets diagnosed. Neave et al. [54] found that cows later diagnosed with metritis spent less time lying and had fewer lying bouts in the two weeks before calving. During the three days before diagnosis, cows with metritis had fewer lying bouts and a longer bout duration, as compared with healthy cows. In addition, cows with metritis spent more time fully standing in the lying stall and had more failed lying events during the three days before metritis diagnosis, when compared to healthy cows [179]. No association between the occurrence of metritis or prepartum activity (measured as arbitrary units) was observed by Liboreiro et al. [145].

Altogether, those studies indicate that metritis may produce visceral pain [174], which may be exacerbated during lying movements. Visceral pain may lead to somatic hyperalgesia (e.g., [180]), making skin areas sensitive to touch during lying movements and when lying. Future studies should benefit from the inclusion of hyperalgesia measurements in dairy cows with metritis.

### 8.2. The Effect of NSAIDs on Activity Patterns in Cows with Metritis

The benefits of NSAIDs as a supportive treatment of metritis in addition to antibiotics show some discrepancies. Several studies reported that cows treated with NSAIDs after metritis diagnosis improve their reproduction performances by showing increasing pregnancy rates [181] and shortened calving-to-first-oestrus intervals [182]. However, carprofen administered alone [183] or NSAIDs administered in conjunction with antibiotics [181] to cows with metritis did not improve milk yield. Several studies also showed divergences in the effects of NSAIDs on uterine involution or recovery from uterine infection [182,184,185,186].

Only one study on lying behaviour reports the effect of meloxicam administered as a complementary treatment to antimicrobial therapy as metritis treatment [187]. In multiparous cows, complementary meloxicam treatment was associated with decreased lying times during five days after treatment, but there was no treatment effect for primiparous cows. Lying bouts duration was not affected by treatment, and the number of lying bouts tended to show a lesser increase in cows treated with meloxicam, as compared with cows that received the placebo [187].

## 9. Concluding Remarks and Potential Further Research

This narrative review shows that pain due to several health conditions in dairy cows can modify daily activity patterns, depending upon the cause, the severity and the moment.

Cows with clinical mastitis reduced their time lying and increased the number of lying bouts and stepping. These activity pattern changes are due to pain caused by the swollen udder when cows are lying and to frustration from not being able to rest as long as necessary. Additional research is needed to observe whether their activity pattern changes with different severity of mastitis (including subclinical mastitis). Two studies have investigated the effect of flunixin meglumine on experimentally induced mastitis and reported that daily activity patterns were not affected by treatment. Additional research including the effect of other NSAIDs on experimentally induced mastitis on lying time includes a closer administration before and after challenge and/or a repeated dose administration. Additional research is guaranteed assessing the effect of NSAIDs on daily activity patterns in naturally occurring mastitis in dairy cows.

Overall, lame cows show longer lying times, with a lower number of lying bouts and longer and more variable lying bouts duration, as compared to non-lame cows. Daily activity patterns are a potential useful indicator for severe lameness but show limitations for detecting moderate lameness. When the relationship between lameness and daily activity patterns is studied, bedding material and the inclusion of severely lame cows are important factors to take into consideration. Two studies using flunixin meglumine and meloxicam confirm that analgesia used in experimentally induced lameness and severe lameness show the welfare of cows is increased by increasing the lying time and decreasing stepping. However, inconsistent or non-clinically relevant outcomes have been reported for other field studies. Additional research on assessing lameness using daily activity patterns is recommended by reducing the clinical heterogeneity of lameness and assessing its degree of severity. Measures aimed to detect central sensitization due to chronic lameness are recommended.

It is assumed that the increased activity around calving is due to pain. Deviation from normal daily activity patterns around calving (either increased or reduced) are indicative of additional pain due to dystocia or a cow delivering a stillborn calf. When pain at calving and daily activity patterns are studied, factors such as parity and the degree of difficulty at calving should be taken into consideration. Additional research studies that include an objective definition of severity of calving (for instance, including physiological indicators of inflammation) are needed. Overall, stepping is altered by NSAIDs treatment around calving, although it shows divergent results among studies. The potential benefits of treating periparturient cows with NSAIDs depend upon the type of drug employed and its dosage and administration mode, as well as the timing of administration relative to calving. NSAIDs that preferentially inhibit COX-2 activity should be preferred for use around calving as they are less likely to have the side effects described.

Primiparous cows with metritis spent more time lying down and have reduced activity, whereas multiparous cows reduced their number of lying bouts. These activity pattern changes are associated with sickness behaviour and avoidance of pain during lying movements. Further research on clinical metritis and daily activity patterns has to take into consideration an objective definition of the severity of the metritis (including whether metritis compromises the cows systemically or only locally) and the inclusion of measures of hyperalgesia. We encourage researchers to perform more studies regarding the effects of the NSAIDs on daily activity patterns in cows with metritis.

When the relationship between painful disorders and daily activity patterns is studied, factors such as parity, bedding type and severity of disease are important factors to take into consideration. In addition, future investigations should include indicators of sickness behaviour and hypersensitivity (hyperalgesia and allodynia) in order to properly interpret daily activity-pattern changes due to painful disorders in dairy cows. The potential benefits of the NSAIDs treatment in painful health disorders depend upon the type of drug administered, its dosage and administration mode, and the time of administration relative to the painful health disorder.

## Figures and Tables

**Table 3:** Summary of changes in activity patterns in cows (lying time, number of lying bouts, lying bout duration, and activity in the sound limb/s) caused by lameness, observing cows with and without analgesia (analgesia vs. control group). Lameness assessment score (from 1 = non-lame to 3 or 5 = severely lame), housing system and bedding type and the moment of analgesia administration are documented. Moment (in days, d, or weeks, wk, related to moment of analgesia administration) and type of change (↑ increase; ↓ decrease; NS = non-significant; NE = non-evaluated; NR = non-reported) are given comparing the analgesia group with control group cows.

Reference	Housing System and Bedding Type	LSC	Criteria of Cows in Pain Due to Lameness	Moment of Analgesia Administration	Analgesia Group	Control Group	Lying Time (h/d)	No. Lying Bouts (No./d)	Lying Bout Duration (min/d)	Activity in the Sound Limb/s (No. Steps/Day)
[121]	Free-stall (mattress)	5-point scale	Score ≥ 3	At the time of hoof trimming	Flunixin meglumine (2.2 mg/kg BW; IV)	Sterile saline solution (4.4 mL)	NS	↓ (wk+3)	↑ (wk+3)	NE
[118]	Free-stall (sand)	5-point scale	Score > 3	After the morning milking	Ketoprofen (3.0 mg/kg BW, IM) 2 consecutive days	Isotonic saline solution (3.0 mg/kg BW)	NS	NS	NS	NS
[128]	NR	4-point scale	Score 2 or 3	After trimming	Ketoprofen (3 mg/kg, IM) once per day for 3 days	Non-lame (score 0 or 1)	NS	NS	NS	NE
			Score 2 or 3	After trimming + foot block (applied to the healthy claw)	Ketoprofen (3 mg/kg, IM) once per day for 3 days	Non-lame (score 0 or 1)	NS	NS	NS	NE
[119]	Free-stall (sand)	5-point scale	Score >3	Before hoof trimming	Flunixin meglumine (2.2 mg/kg of BW) 2 consecutive days	Saline solution (1 mL/22.7 kg BW) 2 consecutive days	↓ (t) (+3D)	NE	NS	NS
[131] ^1^	Free-stall (straw)	5-point scale (with 13 intermediate levels). Septic arthritis of the coffin joint	Score ≥ 9	Before and after surgical section + foot block	Local anaesthesia + Meloxicam (0.5 mg/kg BW, IV) before surgery and once daily from 1 to 4 days	Local anaesthesia + Sterile isotonic saline solution (0.5 mg/kg, IV) before surgery and once daily from 1 to 4 days	↓ (from D+1 to D+7)	NS	↓(t) (from D+1 to D+7)	↑
[129]	Individual stalls (NR bedding)	5-point scale Induced lameness by Amphotericin B (20 mg)	Score > 0	At induced lameness	Flunixin meglumine (1 mg/kg, IV) at the time of induction and 12 h after induction	Nothing	↓ (from induction to D+1)	NE	NE	NE

Significant differences are established at *p* ≤ 0.05, and tendency (t) at *p* ≤ 0.1. LSC = Lameness Scoring System; BW = body weight; IM = intramuscular; IV = intravenous. ^1^ [131] shows the results as total time standing, standing bouts (no./d) and duration of standing bouts (h). For the purpose of the present table, the complementary parameter was included.

**Table 5:** Summary of changes in activity patterns in cows (lying time, number of lying bouts, lying bout duration and activity) caused by calving, observing cows with and without analgesia (analgesia vs. control group). Degree of assistance at calving and moment of analgesia administration are documented. Moment (in days, D; D0 = day of calving) and type of change (↑ increase; ↓ decrease; NS = non-significant; NE = non-evaluated) are given comparing the analgesia group with control group cows. Primiparous cows (PRIM) and multiparous cows (MULT) are mentioned.

Reference	Degree of Assistance at Calving	Moment of Analgesia Administration	Analgesia Group	Control Group	Lying Time (h/d)	No. Lying Bouts (No./d)	Lying Bouts Duration (min/d)	Activity (No. Steps/d)
[157]	Eutocia (cows that calved in ≤70 min) and dystocia (cows that calved in >70 min)	Between 48 h and 6 h before calving	Meloxicam (1 mg/kg, oral)	Placebo (empty gel capsule)	NS	↑ (PRIM, D0)	NS	↓ Jersey (D-1, D+1, D+2) NS Holstein NS (eutocia) ↓ (dystocia)
		Within 12 h after calving	Meloxicam (1mg/kg, oral)	Placebo (empty gel capsule)	NS	NE	NE	↓ Jersey (D+6, D+7) NS Holstein NS (eutocia) ↓ (dystocia)
[146]	Unassisted calving (score 1), assisted by 1 person (score 2), assisted by 2 or more people (score 3) and mechanical assistance (score 4)	Within 12 h after calving	Acetylsalicylic acid (100 mg/kg, oral)	Placebo (gelatin capsules filled with water)	NS	NS	NS	↑ (D+1-D+7)
[135]	Unassisted and easy manual pull	Within 6 h after calving	Meloxicam (0.5 mg/kg, SC)	Placebo (excipient)	NE	NE	NE	↑ PRIM (D+1, D+2) NS MULT
[156]	Easy manual pull (Score 1) and difficult manual pull with more than 1 person and/or mechanical assistance (score 2)	24 h after calving	Meloxicam (0.5 mg/kg, SC)	Placebo (excipient)	NS	NS	NS	NE
[160]	Unassisted and assisted	From 6 h to 10 h after calving	Carprofen (1.4 mg/kg, IV)	Placebo	NE	NS	NE	NE

Significant differences are established at *p* ≤ 0.05. BW = body weight; IM = intramuscular; IV = intravenous.

## Data Availability

Not applicable.
